# Comparison of Chemical Compositions in Pseudostellariae Radix from Different Cultivated Fields and Germplasms by NMR-Based Metabolomics

**DOI:** 10.3390/molecules21111538

**Published:** 2016-11-15

**Authors:** Yujiao Hua, Ya Hou, Shengnan Wang, Yang Ma, Zixiu Liu, Lisi Zou, Xunhong Liu, Yiyuan Luo, Juanxiu Liu

**Affiliations:** College of Pharmacy, Nanjing University of Chinese Medicine, Nanjing 210023, China; huayujiao1020@163.com (Y.H.); yp0317@163.com (Y.H.); jshmwsn@163.com (S.W.); nzymayang1990@163.com (Y.M.); liuzixiu3221@126.com (Z.L.); ggn0117@sohu.com (L.Z.); luoyiyuan0012@sohu.com (Y.L.); liujx0516@163.com (J.L.)

**Keywords:** Pseudostellariae Radix, ^1^H-NMR, metabolomics, cultivated fields, germplasms

## Abstract

Pseudostellariae Radix (PR) is an important traditional Chinese medicine (TCM), which is consumed commonly for its positive health effects. However, the chemical differences of PR from different cultivated fields and germplasms are still unknown. In order to comprehensively compare the chemical compositions of PR from different cultivated fields, in this study, ^1^H-NMR-based metabolomics coupled with high performance liquid chromatography (HPLC) were used to investigate the different metabolites in PR from five germplasms (jr, zs1, zs2, sb, and xc) cultivated in traditional fields (Jurong, Jiangsu, JSJR) and cultivated fields (Zherong, Fujian, FJZR). A total of 34 metabolites were identified based on ^1^H-NMR data, and fourteen of them were found to be different in PR from JSJR and FJZR. The relative contents of alanine, lactate, lysine, taurine, sucrose, tyrosine, linolenic acid, γ-aminobutyrate, and hyperoside in PR from JSJR were higher than that in PR from FJZR, while PR from FJZR contained higher levels of glutamine, raffinose, xylose, unsaturated fatty acid, and formic acid. The contents of Heterophyllin A and Heterophyllin B were higher in PR from FJZR. This study will provide the basic information for exploring the influence law of ecological environment and germplasm genetic variation on metabolite biosynthesis of PR and its quality formation mechanism.

## 1. Introduction

Pseudostellariae Radix (Taizishen in Chinese, PR) is an arid tuberous root of *Pseudostellaria heterophylla* (Miq.) Pax ex Pax et Hoffm (Caryophyllaceae). It is a type of staple traditional Chinese medicine (TCM) which has the functions of strengthening the spleen, replenishing Qi, moistening lungs, and producing fluids. This medicinal herb is consumed clinically for its positive effects [1]. It is reported that PR can be used for inappetence, thirst, debility, diabetes, and weakness after illness, and it has become an important medicine to cure loss of appetite in children due to spleen deficiency [2,3,4,5,6]. As the resources of wild PR are declining and the demand for original medicinal materials is rising annually, the government has established a large-scale cultivation base for PR in Zherong City, Fujian Province; Shibing City, Guizhou Province; and Xuanzhou City, Anhui Province, China, in addition to the traditional growing field in Jurong City, Jiangsu Province. However, due to differences in the ecological environments and expanding cultivated fields, the accumulation of active components and the quality of the material have shown marked differences in PR from various sites. For example, Heterophyllin A and Heterophyllin B were the active components in PR, which showed remarkable differences in different sites, and this may lead to differences in quality of commercial medicinal materials [7]. Thus, it is difficult to implement commodity standardization of medicinal materials and to ensure the effectiveness of its clinical application. Thus, how to ensure the quality and uniformity of PR have become key issues in its production process.

It has been reported that nucleoside, polysaccharide, saponin, pseudostellarins, and heterophyllins are rich in PR [8,9,10,11]. Currently, the quality assessment of PR are mainly focused on the quantitative determination of nucleoside, polysaccharide, saponin, pseudostellarins, and heterophyllins. In Chinese Pharmacopoeia 2010, the content of heterophyllins B was used to assess the quality of PR. However, the components of PR are complex, one or several components cannot assess the quality of this herbal medicine. Metabolomics has recently emerged as an important method for modern research on medicinal plants, and this technology can analyze the whole components in PR, which may fully reflect the immanent quality of medicinal materials. NMR spectroscopy is a key technique used for plant metabolomics due to its non-selectivity, speed, high throughout and relatively easier sample preparation [12]. The ^1^H NMR-based metabolomics approach has been widely applied to reveal metabolic differences among herbal medicines, such as *Forsythia suspense* [13], *Coptis chinensis* [14], *Panax ginseng* [15]. In the present study, ^1^H-NMR-based metabolomics coupled with HPLC quantitative determination were performed to compare the chemical differences and find the change regularity in PR from different cultivated fields and germplasms. The results may provide the basic information for exploring the influence law of ecological environment and germplasm genetic variation on metabolite biosynthesis of PR and its quality formation mechanism.

## 2. Results and Discussion

### 2.1. ^1^H-NMR Metabolic Profiling

#### 2.1.1. Assignment of ^1^H-NMR Spectra

**Figure 1:**
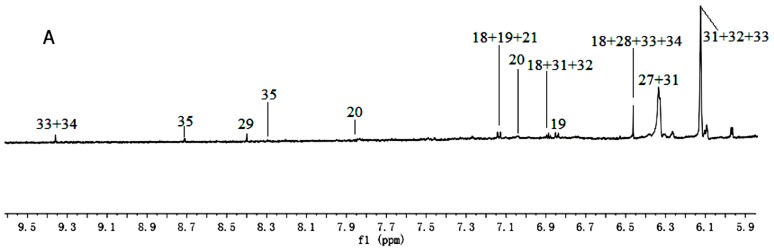

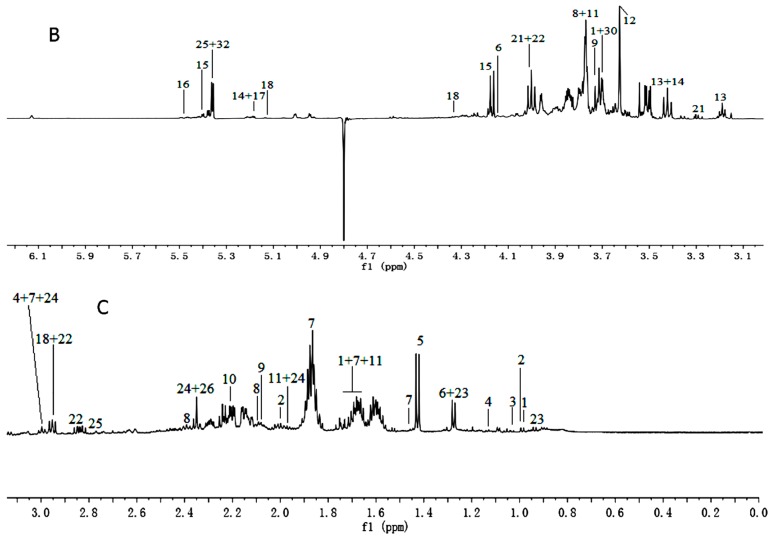
^1^H-NMR spectra (600 MHz) of aqueous methanol fractions in PR from JSJR in low field region (**A**); middle field region (**B**) and high field region (**C**). NMR solvent: CD_3_OD:KH_2_PO_4_ buffer in D_2_O 1:1.

**Figure 2:**
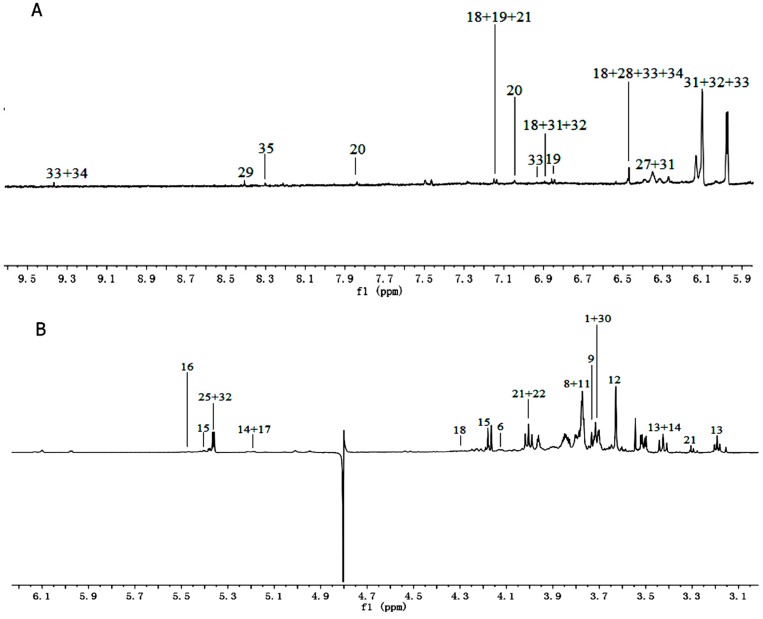

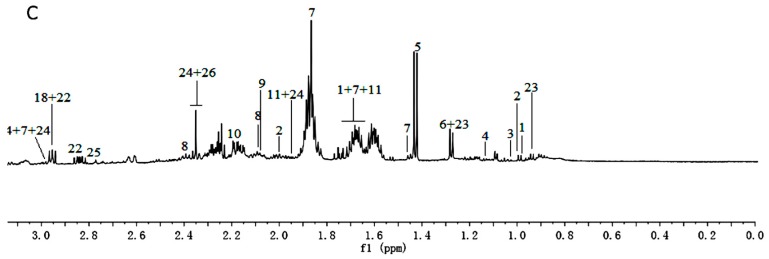
^1^H-NMR spectra (600 MHz) of aqueous methanol fractions in PR from FJZR in low field region (**A**); middle field region (**B**) and high field region (**C**). NMR solvent: CD_3_OD:KH_2_PO_4_ buffer in D_2_O 1:1.

^1^H-NMR spectra signals were assigned based on comparisons with the chemical shift of authentic standards, metabolites in the Biological Magnetic Resonance Data Bank (BMRB) and NMR data in the literature [16,17]. Representative ^1^H-NMR spectra of PR from different cultivated fields were shown in Figure 1 and Figure 2, with metabolites indicated based on their chemical shifts, coupling constants, and peak pattern. A total of 34 metabolites were identified in Table 1. The atlas can be divided into three regions, a high field region (δ 3.10–0.00), consisting of amino acids and organic acids, such as leucine, lysine, alanine, succinate, and 2-Ketoisovaleric acid etc.; a middle field region (δ 6.00–3.10), mainly including carbohydrates, such as α-glucose, sucrose, raffinose, and xylose etc.; and a low field region (δ 10.00–6.00) including fumaric acid, formic acid, quercetin, hyperoside, and luteolin, etc.

#### 2.1.2. Multivariate Data Analysis

**Figure 3:**
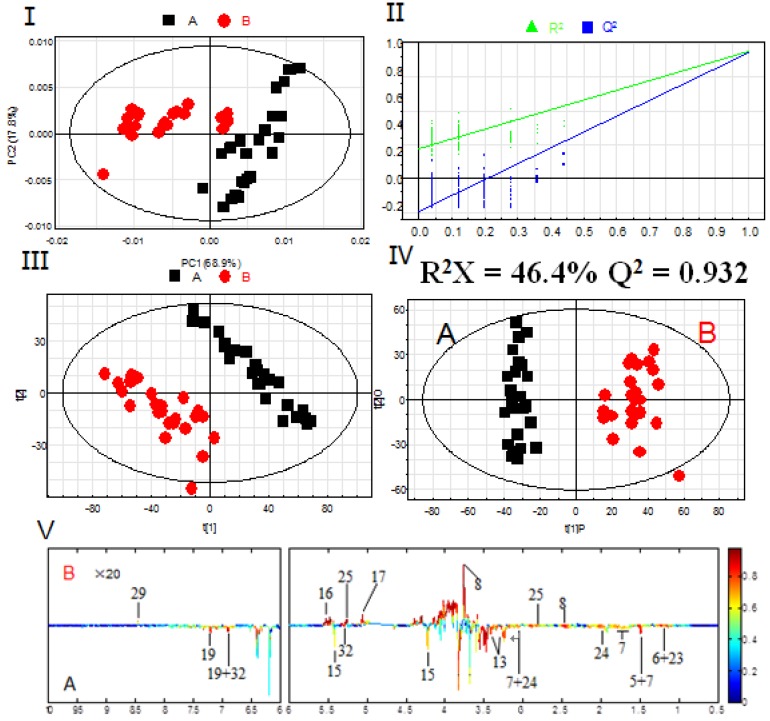
PCA scores plot (**I**); permutation test with 200 permutations of PLS-DA model (**II**); PLS-DA scores plot (**III**); OPLS-DA scores plot (**IV**); correlation coefficients loading plot (**V**) obtained from ^1^H-NMR metabolic profiles derived from aqueous methanol fractions of PR from different cultivation fields. (**A**) JSJR; (**B**) FJZR.

Based on the assignments of ^1^H-NMR spectra, chemical classification of all samples was performed by multivariate data analysis, which aimed to highlight the differences in PR from different cultivated fields. An unsupervised approach consisting of principal component analysis (PCA), a nonparametric method of classification, was used to reduce the dimensions of multivariate problems. The PCA score plot (PC1: 68.9%, PC2: 17.8%), which accounted for 86.7% of total variance of the dataset, showed clear separation in PR from different cultivated fields (Figure 3A). This indicated that the PR from JSJR and FJZR were significantly different in their metabolites, which may be caused by the ecological environment in different cultivated fields. However, the PR samples also showed interclass differences due to the different germplasms, which indicated that the genetic factors can affect the chemical components.

The partial least square discriminant analysis (PLS-DA) extends a regression of PCA and uses class information to maximize the separation between groups of observations. This frequently used classification method is categorical (categories described with dummy variables) and expresses the class membership of the statistical units. In this study, PLS-DA model was also validated by a permutation test with 200 permutations (Figure 3B). The PLS-DA score plot (R^2^X = 46.4%, R^2^Y = 0.947, Q^2^ = 0.930, Figure 3C) showed that the PR from JSJR and FJZR were clearly clustered into two groups, and the interclass differences were less than that in the PCA model, which was more useful to confirm the differences in two different groups.

**Figure 4:**
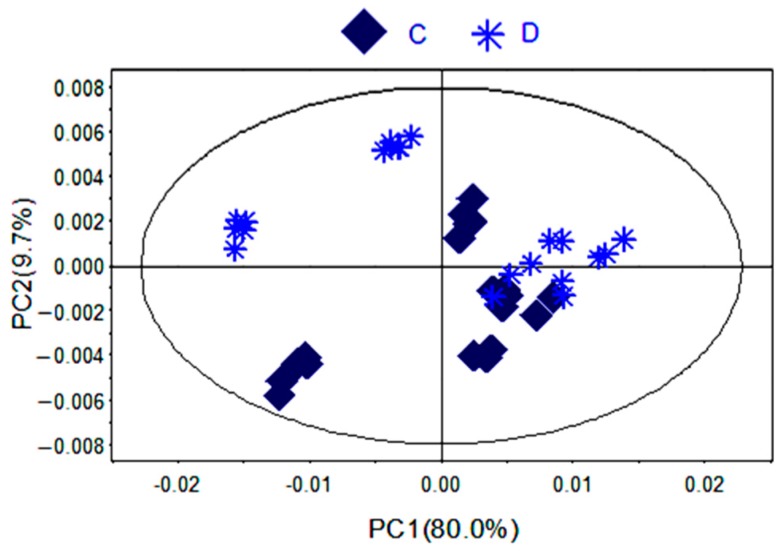
PCA scores plot of PR from different germplasms (**C**) zs; (**D**) jr.

To further identify the significant metabolites contributing to distinction in PR from two different cultivated fields, orthogonal partial least square discriminate analysis (OPLS-DA) of these ^1^H-NMR data was further performed (Figure 3D). Correlation coefficient can directly demonstrate the significantly differential metabolites in PR from different cultivated fields, the plus and minus represented the positive and negative relationship of metabolites. A total of 14 significantly differential metabolites were identified in PR from JSJR and FJZR (Table 2). According to the correlation coefficients loading plot (Figure 3E), the relative contents of alanine, lactate, lysine, taurine, sucrose, tyrosine, linolenic acid, γ-aminobutyrate, and hyperoside in PR from JSJR were higher than that in PR from FJZR. On the contrary, the relative contents of glutamine, raffinose, xylose, unsaturated fatty acid, and formic acid in PR from FJZR were much higher. This indicated that the ecological factor could be an important factor to affect the whole chemical components in the metabolomics of PR. To further demonstrate the ecological factor was the leading factor, the PCA model was used to analyze the PR from jr and zs germplasms which were cultivated in different fields (Jurong City, Jiangsu Province; Zherong City, Fujian Province; Shibing City, Guizhou Province; Xuancheng City, Anhui Province). The results showed that the germplasms had little influence on the whole chemical components in metabolomics of PR (Figure 4).

In fourteen significantly differential components, alanine, sucrose, and linolenic acid were known stress-responsive metabolites. Alanine accumulation in plants in response to exposure to a variety of stress conditions, including sinusoidally-varying magnetic fields (SVMF), is a general phenomenon. The research proposed that alanine is a universal first stress signal expressed by cells [18]. Sucrose is the main form of assimilated carbon which is produced during photosynthesis and then transported from source to sink tissues via the phloem of plants. Additionally, sucrose is engaged in plant defense by activating plant immune responses against pathogens [19]. Linolenic acid is associated with low temperature stress. The study found that the plant stored in 0 °C–Air and 0 °C–CA had much higher degree of linolenic acid content in the plasma membrane than it did that in 5 °C–Air [20].

### 2.2. Content Determination of Heterophyllin A and Heterophyllin B

**Figure 5:**
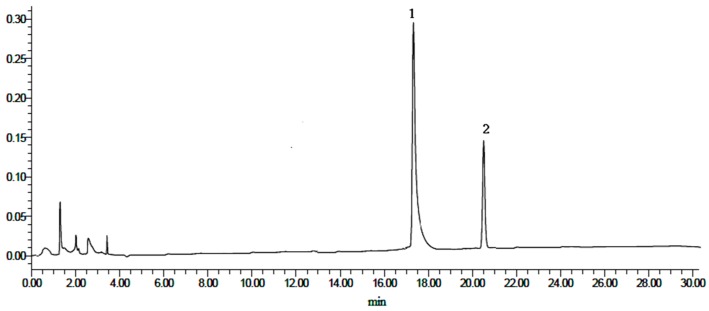
HPLC chromatograms of mixed standards. (**1**) Heterophyllin B, and (**2**) heterophyllin A.

**Figure 6:**
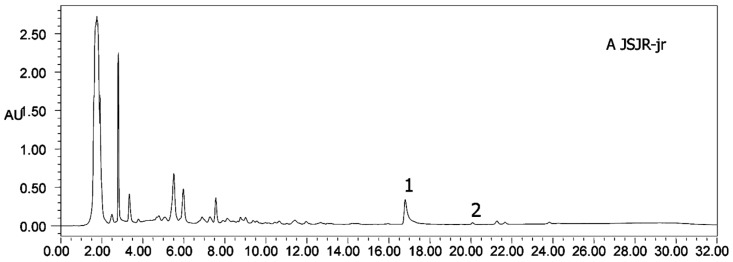

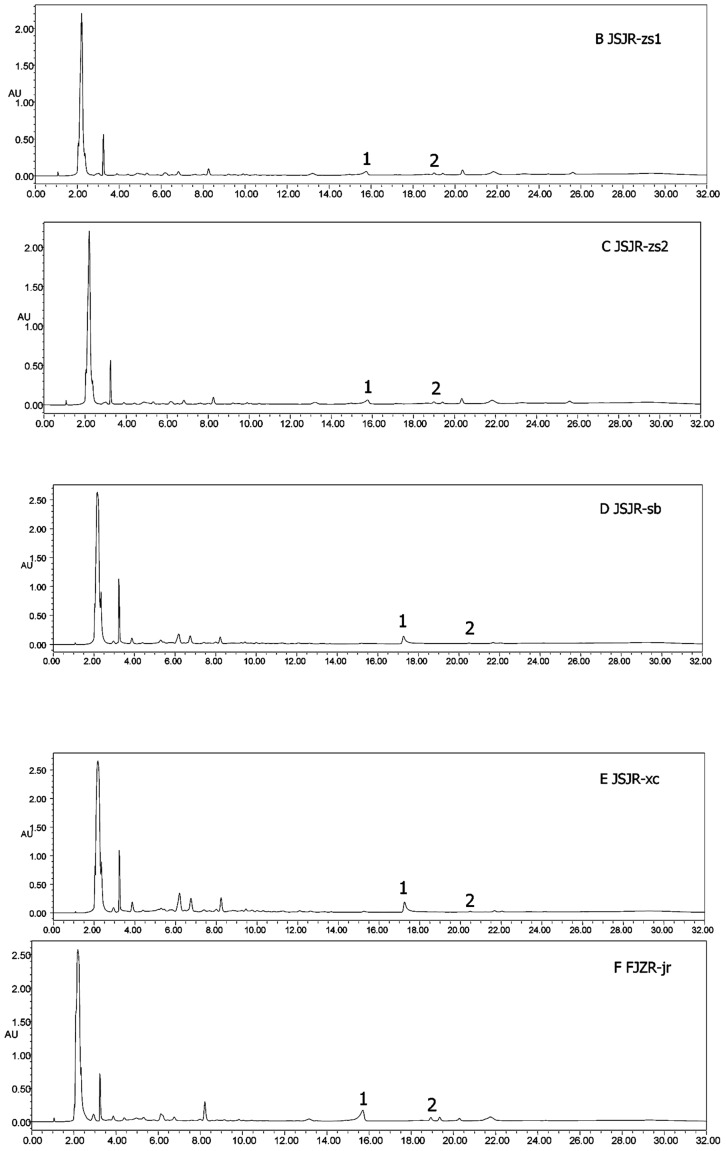

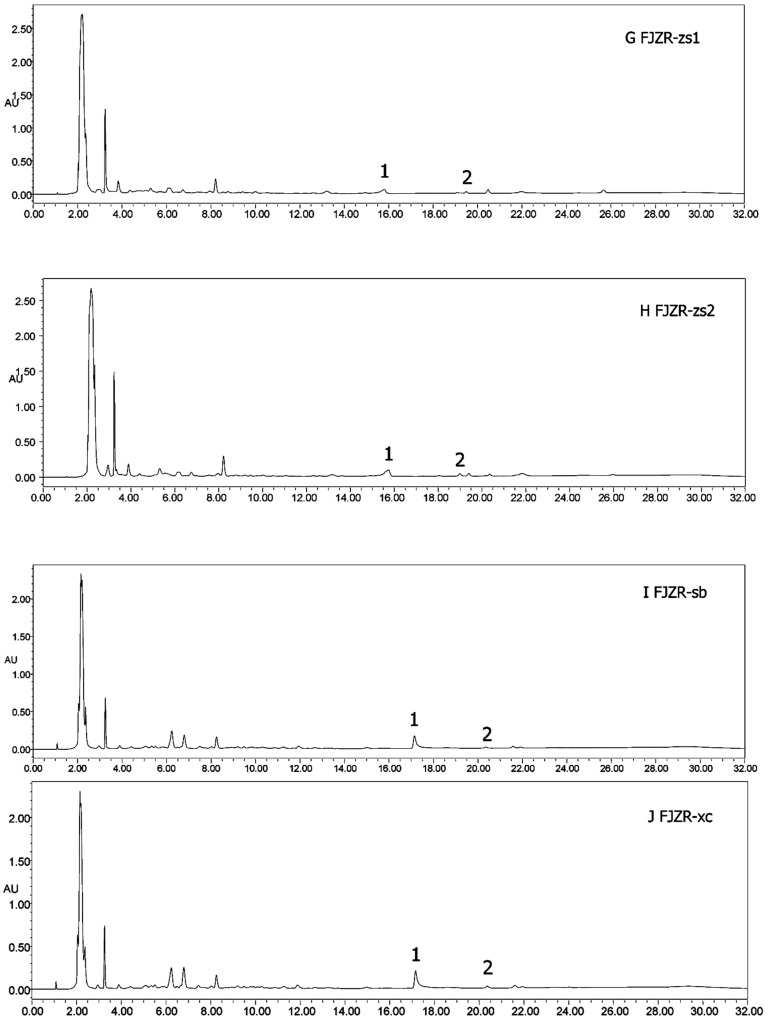
HPLC chromatograms of all samples (A–J). (**1**) Heterophyllin B, (**2**) heterophyllin A.

Heterophyllin A (HA) and Heterophyllin B (HB) are the characteristic components in PR. In Chinese Pharmacopoeia 2010, HB was the index component to assess the quality of PR. In this study, the ^1^H-NMR spectra has not detected these two components; thus, the contents of HA and HB in PR were determined by high performance liquid chromatography (HPLC). The HPLC chromatograms of mixed standards were in Figure 5 and PR samples were in Figure 6. The contents of HA and HB of PR in different cultivated fields were in Table 3. The results showed that the contents of HA and HB in PR from FJZR were much higher than the contents of HA and HB in JSJR. The contents of HB in PR from zs1 and zs2 germplasms were significantly lower than the contents of HB in PR from jr, sb, and xc germplasms; the contents of HA in PR from zs1 and zs2 germplasms were higher than the contents of HA in PR from jr, sb, and xc germplasms. The contents of HA were higher than the contents of HB in PR from zs1 and zs2 germplasms, the contents of HB were higher than the contents of HA in PR from jr, sb, and xc germplasms. This indicated that the genetic factor of germplasms may play an important role in heterophyllins of PR.

## 3. Materials and Methods

### 3.1. Chemicals and Reagents

**Figure 7:**
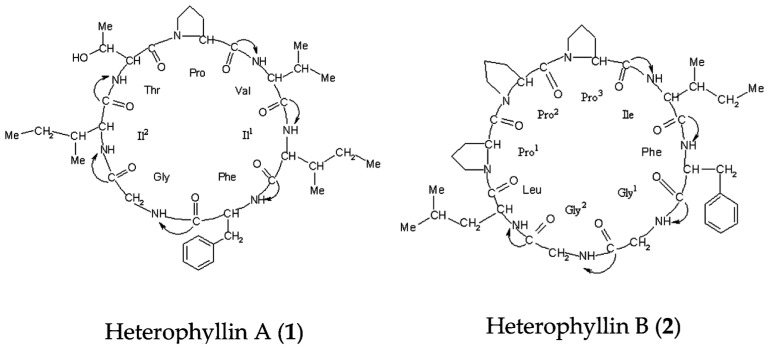
Chemical structures of the two compounds analyzed in the study.

Heterophyllin A (**1**) and Heterophyllin B (**2**) (Figure 7) were provided by Professor Ninghua Tan (Kunming institute of botany, Chinese academy of science, Kunming, China). HPLC analysis showed that their purities were over 98%. Analytical grade methanol and acetonitrile were purchased from Sigma (St. Louis, MO, USA). D_2_O was bought from Norell (Landisville, NJ, USA). Sodium 3-trimethlysilyl [2, 2, 3, 3-d_4_] propionate (TSP) was obtained from Cambridge Isotope Laboratories, Inc. (Andover, MA, USA). KH_2_PO_4_ was from Beijing Chemical Works (Beijing, China), and NaOD was purchased from Armar (Dottingen, Switzerland).

### 3.2. Plant Materials

Five germplasm resources of PR (jr, zs1, zs2, sb, xc) were collected from genuine producing areas (Jurong City, Jiangsu Province; Zherong City, Fujian Province; Shibing City, Guizhou Province; and Xuanzhou City, Anhui Province), and then cultivated in two cultivated bases from Jurong City, Jiangsu Province (JSJR) (119°16′25′′ N, 31°41′15′′ E) and Zherong City, Fujian Province (FJZR) (119°54′2′′ N, 27°13′48′′ E) simultaneously in early November 2012. These samples were harvested in June to August 2013 and were numbered as JSJR-jr, JSJR-zs1, JSJR-zs2, JSJR-sb, JSJR-xc, FJZR-jr, FJZR-zs1, FJZR-zs2, FJZR-sb, FJZR-xc. The samples were authenticated by Prof. Xunhong Liu of Nanjing University of Chinese Medicine. All voucher specimens were deposited in laboratory of identification of Chinese medicine. All samples were ground to a fine powder and sieved with a bolt (80 mesh). The powder was then kept in an airtight container at −80 °C until use.

### 3.3. ^1^H-NMR Measurement

For ^1^H-NMR analysis, all powder samples (100 mg each) were transferred into centrifuge tubes. Methanol (75%, 0.6 mL, water:methanol = 1:2, *v*/*v*) was added to the tube, followed by vortexing for 1 min and ultrasonication for 30 min. The materials were then centrifuged at 3500 rpm for 25 min. The supernatants were transferred separately into a 25-mL round-bottomed flask to dry in a rotary vacuum evaporator. The extraction procedure was repeated three times. Samples were dissolved in 600 µL mixure (1:1) of CD_3_OD and KH_2_PO_4_ buffer in D_2_O (adjusted to pH 6.0 by 1 N NaOD) containing 0.05% TSP. The samples were then centrifuged for 10 min at 13,000 rpm, and supernatants (550 μL) were transferred into 5-mm tubes for NMR analysis. 

^1^H-NMR was recorded at 25 °C on a Bruker 600 MHz AVANCE III NMR spectrometer (Bruker, Karlsruhe, Germany) operating at a proton NMR frequency of 599.83 MHz. CD_3_OD was used for internal lock purposes. Each ^1^H-NMR spectra consisted of 128 scans requiring 10 min acquisition time with following parameters: 0.16 Hz/point, pulse width (PW) = 30° (11.3 µs), and relaxation delay (RD) = 2 s. A presaturation sequence was used to suppress the residual H_2_O signal with low-power selective irradiation at the H_2_O frequency during the recycle delay. Free induction decays (FIDs) were Fourier transformed with Luria–Bertani (LB) = 0.3. The resulting spectra were manually phased and baseline-corrected, and calibrated to TSP at 0.00 ppm. The metabolite data were deposited to MetaboLights [21] with identifier MTBLS399.

### 3.4. Data Analysis

The ^1^H-NMR spectra were processed using MestReNova software (Version 6.1, Mestrelab Research, Santiago de Compostela, Spain), the FID signal was made by Fourier transformation to improve the signal to noise ratio, spectral intensities were scaled to TSP and reduced to integrated regions of equal width (0.002 ppm) according to the region of δ 0.50–10.00. The two regions of δ = 3.355−3.363 and 4.67–4.98 were excluded from analysis because of the residual signals of methanol and water. The integral data (δ = 0.50 to δ = 10.00, except for δ = 3.355−3.363 and 4.67–4.98) were normalized according to peak areas and imported to Microsoft office Excel (Redmond, WA, USA) for the following analysis. The integral data obtained were imported to SIMCA-P 11.5 (Umetrics, Umea, Sweden) for multivariate statistical analysis, including principal component analysis (PCA), partial least squares discriminant analysis (PLS-DA), and orthogonal PLS-DA (OPLS-DA) with VIP > 1.

### 3.5. Content Determination

All PR samples were weighed exactly to 0.0001 g, and each sample was repeated three times. The content determination of HA and HB in PR was referred to the reliable method developed by our research group [7].

## 4. Conclusions

In this study, we first conducted the metabolic profiling using NMR for the analysis of PR. A total of 34 metabolites were identified based on ^1^H-NMR data, and 14 of them were significantly different in PR from different cultivated fields. The relative contents of alanine, lactate, lysine, taurine, sucrose, tyrosine, linolenic acid, γ-aminobutyrate, and hyperoside in PR from JSJR were higher than that in PR from FJZR, while PR from FJZR contained higher levels of glutamine, raffinose, xylose, unsaturated fatty acid, and formic acid. The contents of HA and HB were higher in PR from FJZR.

The chemical components in metabolomics of TCM are very complex, and the heterophyllins have not been detected in this study. In the further experiment, “add standard qualitative test” will be used to explore the complete assignment and complex chemical components in metabolomics with other analytical techniques. This study will provide the basic information for exploring the influence law of the ecological environment and germplasm genetic variation on metabolite biosynthesis of Pseudostellariae Radix and its quality formation mechanism.

## Figures and Tables

**Table 1:** Metabolites detected by ^1^H-NMR in PR from different cultivated fields.

No.	Metabolites	Chemical Shift for Standards (δ, ppm), Coupling Constants (*J*, Hz)	Chemical Shift (δ, ppm), Coupling Constants (*J*, Hz)	VIP
1	Leucine	0.95 (t, 5.9), 1.72 (m)	0.97 (d, 7.1), 1.72 (m), 3.71 (s)	0.32
2	Isoleucine	0.926 (t, 7.414), 0.997 (d, 7.001), 1.248 (m), 1.457 (m), 1.968 (m), 3.66 (d, 3.969)	1.02 (t, 7.6), 1.97 (m)	1.09
3	Valine	0.976 (d, 7.01), 1.029 (d, 7.05), 2.261 (m), 3.601 (d, 4.33)	1.05 (d, 6.8)	1.17
4	2-Ketoisovaleric acid	1.15 (m), 3.02 (m)	1.13 (d, 7.2), 3.03 (m)	1.02
5	Alanine	1.47 (d, 7.2), 3.8 (m)	1.43 (d, 7.3)	1.38
6	Lactate	4.1 (s), 1.3 (s)	1.26 (s), 4.10 (s)	1.01
7	Lysine	1.46 (m), 1.7 (m), 1.89 (m), 3.02 (t, 6.09)	1.44 (m), 1.72 (m), 1.87 (m), 3.04 (t, 6.6)	1.31
8	Glutamine	2.12 (m), 2.45 (m), 3.76 (t, 6.18)	2.12 (m), 2.37 (m), 3.76 (m)	1.61
9	Glutamate	2.04 (m), 3.75 (dd, 7.186, 4.72), 2.34 (m)	2.07 (m), 2.34 (m), 3.74 (m)	0.11
10	Acetoacetate	2.27 (s), 3.43 (s)	2.22 (m)	1.51
11	Arginine	1.68 (m), 1.90 (m), 3.22 (t, 6.93), 3.76 (t, 6.11)	1.72 (m), 1.92 (m), 3.75 (t, 6.1)	1.39
12	Glycine	3.54 (s)	3.61 (s)	1.47
13	Taurine	3.25 (t, 6.57), 3.42 (t, 6.62)	3.40 (t, 12.0), 3.27 (t, 10.2)	1.06
14	α-Glucose	5.2 (d, 3.7)	3.40 (m), 5.19 (d, 3.7)	0.36
15	Sucrose	5.42 (d, 3.6), 4.19 (d, 8.4)	5.40 (d, 3.9), 4.18 (d, 3.9)	1.53
16	Raffinose	4.97 (d, 3.6), 5.43 (d, 4.2)	5.49 (d, 3.8)	1.33
17	Xylose	4.55 (d, 9)	5.19 (m)	0.36
18	Salvianolic acid B	7.15 (d, 8.8), 6.80 (d, 7.2), 6.50 (d, 2.8), 2.95 (m)	7.14 (d, 8.2), 6.85 (d, 8.6), 6.47 (d, 1.8), 5.13 (m), 4.29 (d, 4.8), 2.94 (m)	1.05
19	Tyrosine	6.83 (d, 8.0), 7.15 (m), 6.87 (m), 7.17 (m)	7.12 (t, 8.4), 6.83 (t, 6.6)	1.35
20	Histidine	7.09 (d, 0.58), 7.9 (d, 1.13)	7.10 (d, 8.4), 7.90 (m)	0.89
21	Phenylalanine	3.19 (m), 3.98 (dd, 7.88, 5.31), 7.32 (d, 6.96), 7.36 (m), 7.42 (m)	3.28 (m), 3.99 (m), 7.14 (d, 8.2)	1.53
22	Asparagine	2.94 (m), 2.84 (m), 4.00 (dd, 7.69, 4.2)	2.88 (dd, 16.1, 7.6), 2.96 (dd, 16.1, 7.6), 4.00 (m)	0.52
23	Linolenic acid	0.98 (t, 9.8)	0.96 (t, 6.0), 1.30 (brs)	1.07
24	γ-aminobutyrate	1.94 (m), 2.48 (t, 7.36), 3.03 (t, 7.58)	1.92 (m), 2.32 (t, 9.6), 3.04 (t, 6.6)	1.35
25	Unsaturated fatty acid	2.85 (m), 5.30 (m)	2.75 (m), 5.34 (m)	1.06
26	Succinate	2.39 (s)	2.32 (s)	0.81
27	Ferulic acid	7.15 (d, 8.4), 6.32 (m)	7.14 (d, 8.2), 6.34 (m)	1.53
28	Fumaric acid	6.55 (s)	6.47 (s)	1.69
29	Formic acid	8.32 (s)	8.40 (s)	1.43
30	Dimethylglycine	2.95 (s), 3.75 (s)	2.94 (s), 3.71 (s)	1.12
31	Quercetin	6.18 (d, 2), 6.39 (d, 2), 6.88 (d, 8.5), 7.52 (dd, 2.2, 8.5), 7.66 (d, 2.2), 12.4 (s)	6.85 (d, 8.6), 6.34 (d, 4.9), 6.13 (d, 1.8)	1.14
32	Hyperoside	6.90 (d, 7.2), 5.40 (d, 4.2)	6.85 (d, 8.6), 6.13 (d, 1.6), 5.36 (d, 3.9)	1.14
33	Luteolin	6.4 (d, 2), 6.68 (s), 6.76 (d, 2), 6.89 (d, 9), 7.39 (d, 2), 7.41 (dd, 2.2, 9.0)	6.89 (d, 8.6)	1.32
34	Kaempferol	8.05 (m), 6.5 (d, 2.0), 6.98 (m), 6.25 (d, 2.0)	6.90 (d, 9.0), 6.47 (d, 1.2)	1.69

**Table 2:** Significant differences in the chemical compositions and the correlation coefficient.

No.	Metabolites	Correlation Coefficient
5	Alanine	−0.893
6	Lactate	−0.865
7	Lysine	−0.648
8	Glutamine	+0.924
13	Taurine	−0.805
15	Sucrose	−0.645
16	Raffinose	+0.950
17	Xylose	+0.858
19	Tyrosine	−0.690
23	Linolenic acid	−0.589
24	γ-Aminobutyrate	−0.631
25	Unsaturated fatty acid	+0.707
29	Formic acid	+0.674
32	Hyperoside	−0.677

**Table 3:** Contents of Heterophyllin A and Heterophyllin B of PR in different cultivated fields. Μg·g^−1^, *n* = 3.

No.	Heterophyllin A	Heterophyllin B
JSJR-jr	9.32	138.42
JSJR-zs1	36.32	2.54
JSJR-zs2	16.22	3.16
JSJR-sb	6.88	65.47
JSJR-xc	6.20	130.69
FJZR-jr	14.99	175.58
FJZR-zs1	43.73	2.75
FJZR-zs2	28.09	5.02
FJZR-sb	12.18	96.01
FJZR-xc	11.55	149.27
